# The scientific nature of the linear no-threshold (LNT) model used in the system of radiological protection

**DOI:** 10.1007/s00411-024-01092-1

**Published:** 2024-09-02

**Authors:** Andrzej Wojcik, Friedo Zölzer

**Affiliations:** 1 Centre for Radiation Protection Research, MBW Department, Stockholm University, Stockholm, Sweden; 2 Institute of Biology, Jan Kochanowski University, Kielce, Poland; 3 Institute of Radiology, Toxicology and Civil Protection, Faculty of Health and Social Sciences, University of South Bohemia in Ceske Budejovice, Ceske Budejovice, Czech Republic

**Keywords:** LNT, Low radiation dose, Radiological protection, Precautionary principle, ICRP, Dose limitation, Scientific basis, Ethical basis

## Abstract

During the first half of the 20th century, it was commonly assumed that radiation-induced health effects occur only when the dose exceeds a certain threshold. This idea was discarded for stochastic effects when more knowledge was gained about the mechanisms of radiation-induced cancer. Currently, a key tenet of the international system of radiological protection is the linear no-threshold (LNT) model where the risk of radiation-induced cancer is believed to be directly proportional to the dose received, even at dose levels where the effects cannot be proven directly. The validity of the LNT approach has been questioned on the basis of a claim that only conclusions that can be verified experimentally or epidemiologically are scientific and LNT should, thus, be discarded because the system of radiological protection must be based on solid science. The aim of this publication is to demonstrate that the LNT concept can be tested in principle and fulfils the criteria of a scientific hypothesis. The fact that the system of radiological protection is also based on ethics does not render it unscientific either. One of the fundamental ethical concepts underlying the LNT model is the precautionary principle. We explain why it is the best approach, based on science and ethics (as well as practical experience), in situations of prevailing uncertainty.

## Introduction

A basic assumption of the international system of radiological protection, as recommended by the International Commission on Radiological Protection (ICRP), is that the risk of radiation-induced cancer is directly proportional to the dose received, without any dose level (threshold) below which the risk is zero (ICRP_99 2005). This so-called linear no-threshold (LNT) model is strongly criticized by, on the one hand, researchers claiming that it underestimates the actual risk, because it does not consider, among other possible modifying factors, bystander effects according to which the relationship is more properly described by a supralinear curve (Mothersill and Seymour 2004). On the other hand, some researchers claim that adaptation processes reduce the radiation-related risk at low doses, resulting in a threshold dose below which there is either no effect or even health benefit (hormesis) (Sacks et al. 2016; Janiak and Waligorski 2023). Yet others claim that both mechanistic evidence coming from radiobiology and observational evidence coming from epidemiology suggest that a dose threshold, if any, could not be greater than a few tens of mGy and, thus, the LNT model has a solid basis in results from experimental studies (Laurier et al. 2023). Also, UNSCEAR in a review of biological data, concluded that there remains good justification for the use of a non-threshold model for risk inference given the robust knowledge on the role of mutation and chromosomal aberrations in carcinogenesis (UNSCEAR 2021).

Why is it not possible to reach a consensus regarding the shape of the dose response? At low radiation doses, defined as below 0.1 Gy (UNSCEAR 2012), biological effects are very weak so they are easily influenced by random environmental factors making results difficult to reproduce. A good example are the variable results of adaptive response experiments (Wojcik and Streffer 1994; Wojcik et al. 1996; Wojcik and Shadley 2000). In general, despite new, suggestive epidemiological data (Laurier et al. 2023), mechanistic, experimental approaches with both cell and animal models are unable to provide unequivocal evidence for the existence of a dose threshold below which radiation carries no risk to human health. There is still insufficient knowledge about the sequence of events from the deleterious alteration of biomolecules to the diagnosable disease, i.e. to stochastic cancer or non-cancer effects (UNSCEAR 2021). Also, epidemiological studies mostly lack the necessary statistical power to detect effects at doses below 0.1 Gy (Ruhm et al. 2022).

Disagreement about the interpretation of results is an essential element of science and many famous scientific discoveries were accompanied by controversy and disputes (Sarewitz 2011). Consequently, it is desirable that the debate around LNT continues. However, its validity has been questioned on the basis that it is not a scientific concept and should thus be discarded because the system of radiological protection must be based on solid science (Waltar et al. 2023). This line of argumentation precludes any constructive debate: no researcher will waste time on a non-scientific concept. More importantly, it is flawed because the system of radiological protection, as designed by the ICRP, is “based on scientific knowledge, ethical values, and more than a century of practical experience” (ICRP_138 2018). It relies wholly on state-of-the art science, understood broadly as knowledge (as distinguished from ignorance or misunderstanding), and the LNT model, being an element of the system, is a scientific concept. The aim of this publication is to demonstrate this.

## LNT as a scientific concept

The authors of the recent LNT critique (Waltar et al. 2023) write that LNT lacks a solid scientific base because there are no “actually proven radiation effects at low-doses”. The risk of cancer induced by high doses of radiation, they argue, can be derived from frequentist probabilities which “are based on evidence; namely, on the truthful and verifiable existence of an increase in the frequency of radiation health effects in a cohort of exposed people and are defined as the limit of the relative frequency of incidence of the effect in a series of certifiable epidemiological studies on such cohorts”. In contrast, the risk of cancer in the low dose region is assessed based on “subjective probabilities (sometimes also confusedly termed “Bayesian”), which are conjectured for the low-dose area, expressed as a possible expectation that radiation health effects might occur, and are quantified by a personal belief or expert’s judgement; that is, not necessarily substantiated by the frequency or propensity that the effects actually occur at such levels of dose”. In short: the assumption of no threshold dose for the risk of cancer is not scientific because it cannot be proven.

The claim that whatever cannot be proven in experimental or epidemiological studies is not scientific may be based on a particular reading of Karl Popper´s “critical rationalism”, which suggests a method to distinguish between science and non-science. But if the authors had that approach in mind, their reading of it is wrong: lack of proof does not, in Popper’s view, make a hypothesis unscientific. On the contrary, Popper maintains that nothing whatsoever can really be proven; there are no verifiable truths. What scientists can do is to test a given hypothesis over and over again. If they find satisfactory evidence against it, the hypothesis is “falsified”. If they do not find evidence against it, it is “corroborated”. The more “corroboration” we have, the more certain we can be of that particular hypothesis, but we can still not consider it “verified”. So, the criterion of “scientific” vs. “unscientific” is not “verifiability”, but “falsifiability” (Popper 1961). Popper´s method has been severely criticized for reasons that will not be discussed here. The interested reader is referred to relevant publications (Maxwell 1972; Feyerabend 2010). Despite this ongoing discussion in science theory, however, there is no doubt that the lack of positive proof for a certain model does not render it unscientific. This is true for the LNT model as well. It can, in principle, be tested. For instance, recent epidemiological studies with large numbers of people undergoing medical radiology did not show any indication of a threshold for cancer induction by radiation (Laurier et al. 2023). The latest addition to this growing body of evidence is the EPI-CT study, in which almost a million children who had to undergo a CT examination were followed for several years and their risk of hematological malignancies was quantified. A significant increase was found in the dose group of 10–15 mGy (Bosch de Basea Gomez et al. 2023). With even larger numbers, and more precise and consistent methods of dosimetry as well as diagnosis of disease, it will be possible to corroborate the LNT model even more convincingly. Of course, this will always apply to particular dose ranges and particular effects, but with those caveats in mind, the model can certainly be considered “falsifiable” and, therefore, scientific. To summarize: epidemiological studies have, until now, not been able to falsify LNT (Laurier et al. 2023). Let us have a look at attempts to falsify LNT by other approaches.

## Conclusions from UNSCEAR reports on the shape of the dose response for cancer and derivation of dose limits by the ICRP

In developing its recommendations, the ICRP relies on results from the field of natural science on mechanisms and levels of health effects induced by ionising radiation. These are regularly summarised by UNSCEAR (www.unscear.org). As stated above, the epidemiological evidence on the shape of the dose response curve in the dose range relevant for planned exposure scenarios of people does not falsify LNT, but does not allow drawing firm conclusions due to lack of statistical power. Since 1994, UNSCEAR has published four reports that look into biological effects induced by low dose exposure, with the aim of examining whether they support the assumption of the LNT concept. The 1994 report focused on adaptive responses in cells, experimental animals and humans and concludes that evidence does not exist to support the assumption that adaptive responses convey beneficial effects to the organism that would outweigh the detrimental effects of exposure to radiation (UNSCEAR 1994). The 2000 report did not specifically focus on adaptive responses but aimed at providing an overview of data available on the relationship between radiation exposure and the induction of cancer and hereditary disease (UNSCEAR 2000). It concludes that, although mechanistic uncertainty remains, studies on DNA repair and cellular/molecular processes of radiation tumorigenesis provide no good reason to assume that there will be a low-dose threshold for the induction of tumours in general. In support of this, the authors of the report discuss DNA double strand breaks (DSB) originating from single ionizing tracks of radiation that occur in the low dose range. Although their incidence is low, they may arise from the more likely single strand lesions, when these occur in close proximity on opposed DNA strands. Furthermore, the report points out that single ionization tracks were shown to induce locally multiply damaged sites (LMDS). LMDS pose a particular problem for the cellular DNA repair system and will most likely be misrepaired, leading to a mutation and potentially – cancer. This evidence is important in view of existing opinions that low doses of radiation merely increase the level of the naturally occurring oxidative damage that has no negative consequences because cells are well equipped to cope with it (Tubiana 2005). Of course, oxidative damage does occur naturally and appropriate repair processes exist, but its spatial distribution is different from that caused by the ionisation tracks of photons and particles in the form of locally multiply damaged sites. The aim of the next report, published in 2006, was “to evaluate how non-targeted effects may affect risks associated with radiation exposure, the understanding of radiation-induced carcinogenesis, and the mechanistic basis for interpreting epidemiological data on radiation effects” (UNSCEAR 2006). The report concludes that data currently available do not require changes in radiation risk coefficients for cancer and hereditary effects of radiation in humans. The last report was published in 2021 (UNSCEAR 2021). Its focus is on biological mechanisms of radiation actions at doses mostly in the low to moderate range relevant for cancer risk inference. Consequently, it looks at available knowledge on DNA damage and repair, chromatin remodelling and epigenetics, gene and protein expression, non-targeted effects, the immune system and modelling of cancer mechanisms. In accordance with the previous reports, it concludes that accumulated knowledge on mechanisms of effects directly related to cancer induction imply a dose-risk relationship without a threshold at least down to 10 mGy and that “little in the way of robust data could be identified that would prompt the need to change the current approach taken for low-dose radiation cancer risk inference as used for radiation protection purposes and for the purpose of comparison with other risks”. In summary, neither epidemiological nor mechanistic studies provide unequivocal evidence for the shape of the dose-response curve, although they confirm that the LNT concept is falsifiable in principle – at least for certain dose ranges.

## The ethical basis of radiological protection as a scientific concept

If it is not possible to quantify the risk of stochastic effects at low doses, how did the ICRP arrive at the dose limits that are currently recommended? A historical reconstruction of the considerations underlying the setting of dose limits was recently published by one of us (Zolzer 2022). Here, as well as elsewhere in radiological protection, assumptions about risks at small doses need to be made. If recommendations for radiological protection would have to be based on scientific evidence alone, one might point to the (undeniable and undenied) uncertainties about the LNT concept and remain doubtful as to its applicability. As stated above, however, the ICRP’s system is “based on scientific knowledge, ethical values, and more than a century of practical experience” (ICRP_138 2018). Usually, of course, ethics and practice per se are not considered scientific (which in itself is open to debate), but it needs to be emphasised that the role which they play for the system of radiological protection does not render that system unscientific.

“Ethics” can mean different things. It can denote a set of beliefs and values regarding what is right and what is wrong, and as such can be used in combinations like “the ethics of a particular individual”, “the ethics of a particular group”, or “the ethics of a particular society, culture, or religion“. The same word, however, can also designate a branch of philosophy, sometimes called “moral philosophy”, which systematically studies this kind of beliefs and values. “Ethics” in this sense is clearly a rational endeavour. It examines standards of rightness and wrongness, and their application to practical problems, but it does not single out a concrete standard, i.e. it does not become prescriptive. What is right and what is wrong can only be established within the context of a particular ethical system. Utilitarian ethics, for instance, recognizes as the criterion of right and wrong nothing but the “greatest happiness of the greatest number” (Bentham 1776), whereas in deontological ethics everything depends on “treating humanity, whether in your own person or that of another, never merely as a means to an end” (Kant 1785). There are other systems, of course. Virtue ethics, for instance, has recently received renewed interest (Aristotle being an early proponent). It is concerned not so much with actions and their consequences, but with people’s characters and dispositions. Consistent ethical judgement is possible on the basis of either of these theories, but they do not always lead to the same result. Thus, there is no such thing as an ethics which is universally applicable and binding for all.

Coming back to radiological protection, it may be interesting to note that around the turn of the 21st century several authors, among them members of ICRP, argued that the three principles of radiological protection – justification, optimization, and dose limitation – are based on one or the other classical theory of Western moral philosophy as outlined above. The ICRP itself in its first publication fully dedicated to the topic of ethics (ICRP_138 2018) has discussed this kind of arguments in an appendix to that publication. In the main body of the report, however, they took a different approach. Recognizing that radiological protection is a world-wide endeavour, it was decided to take as a point of departure a certain set of moral values which are common (or at least acceptable) to people from different cultural backgrounds. These values did not have to be invented from scratch but had been referred to implicitly or explicitly in earlier publications of the IRCP.

The approach is similar to an ethical theory suggested in 1979 by Beauchamp and Childress and widely applied in medicine, called the “principles of biomedical ethics” (Beauchamp 1979). The authors originally worked on quite different basic assumptions, one being a utilitarian, the other a deontological ethicist, but they realised that in spite of belonging to different schools of thought, they could still agree on a number of “principles” which allowed them to solve most ethical dilemmas in clinical practice. They identified these principles as Respect of autonomy, Non-maleficence, Beneficence, and Justice. All of them, they maintained, had *prima facie* validity, i.e. all of them seem applicable at first sight without any particular ranking, but in certain clinical situations not all of them can be applied in the same way, one or the other having to take precedence. This they called “balancing the principles” and they discussed many examples of how to determine the relative importance of each principle in particular situations.

What the ICRP proposed in Publication 138 is very similar, but it does not copy the Beauchamp and Childress approach one-to-one. The fundamental concepts are called “values” instead of “principles”, because that term is already used for justification, optimization and dose limitation, and more importantly, the four “core values” are slightly different: Beneficence/Non-maleficence, Prudence, Justice, and Dignity. Their application in different contexts, as well as the necessity to “balance” them against each other, is discussed in Publication 138, as well as Publication 153 on “Radiological Protection in Veterinary Practice” and in the up-coming publication on “Ethics in Radiological Protection for Medical Diagnosis and Treatment”. We will not go into any detail here, but just state again that the ethics of radiological protection, as practiced by the ICRP, is not a promotion of subjective convictions or preferences, or a reflection of “personal beliefs”, but is well in line with current trends in moral philosophy.

Prudence is not part of the Beauchamp and Childress set of principles, or values, but does play an important role for radiological protection. The ICRP itself has pointed this out. The 1956/57 amendment to the 1954 recommendation (ICRP_^1958^, ^1958^) already stated that ‘it is prudent to limit the dose of radiation received by gametes (…) to an amount of the order of the natural background’, and a similar statement appeared in Publication 1 (ICRP_1 1959), where prudence again played an important role in the justification of dose limits. The ICRP recognised that its recommendations could no longer be based on well-documented tissue reactions, but had to take account of stochastic effects for which there was no more than a certain plausibility. And even though the risks were hard to quantify for the time being, one had to make an attempt to weigh them against the expected benefits of activities involving radiation exposure. This is why ICRP recommended early on ‘that every effort be made to reduce exposure (…) to the lowest possible level’ (ICRP_^1955^, ^1955^), ‘that all doses be kept as low as practicable’ (ICRP_1 1959), or ‘that all doses be as low as readily achievable, economic and social consequences being taken into account’ (ICRP_9 1966). All three formulation are obviously early formulations of the ALARA principle, “doses should all be kept as low as reasonably achievable, taking into account economic and societal factors” (ICRP_103 2007). ICRP also suggested that dose limitation ‘necessarily involves a compromise between deleterious effects and social benefits’ (1959) (ICRP_1 1959) and that one has to find ‘a level at which the assumed risk is deemed to be acceptable to the individual and to society in view of the benefits derived from such activities’ (ICRP_9 1966).

In the latest general recommendations (ICRP_103 2007), the ICRP states that “it is prudent to take uncertainties (…) into account”, even when it comes to the estimates of threshold doses for deterministic effects. More importantly, the “so-called linear-non-threshold (LNT) model is considered by the Commission to be the best practical approach to managing risk from radiation exposure… The Commission considers that the LNT model remains a prudent basis for radiological protection at low doses and low dose rates.” Furthermore, in spite of lacking evidence in humans for radiation effects on offspring and next generations, “the Commission prudently continues to include the risk of heritable effects in its system of radiological protection” and “considers that it is prudent to assume that life-time cancer risk following in-utero exposure will be similar to that following irradiation in early childhood, i.e., at most, about three times that of the population as a whole.” In Appendix A, the stress is again on practicality: “The LNT model is not universally accepted as biological truth, but rather, because we do not actually know what level of risk is associated with very-low-dose exposure, it is considered to be a prudent judgement for public policy aimed at avoiding unnecessary risk from exposure.”

In one of the passages just quoted, the ICRP mentions that its emphasis on prudence is “commensurate with the ‘precautionary principle’ (UNESCO 2005)” (for further information on the principle, see (Martuzzi and Bertollini 2004; Tallacchini 2005). This has raised red flags for some, who tend to think that radiological protection is overdone anyway and actually “crippling the beneficial effects that controlled radiation offers to a modern society” (Waltar et al. 2023). It cannot be emphasized enough, however, that the precautionary principle is often (willingly or unwillingly) misinterpreted. It does not say that with the slightest suspicion of a risk, however small it may be, all related activities should be stopped. It does not, as some have put it, provide blanket authorization for technophobia. One of the most widely used versions of the principle states: “When an activity raises threats of harm to human health or the environment, precautionary measures should be taken even if some cause and effect relationships are not fully established scientifically.” This is the so-called ”Wingspread Statement”, issued in 1998 by a diverse group of scientists, philosophers, lawyers and environmental activists from the United States, Canada and Europe. The wording is similar to that of the “Rio Declaration” six years earlier, which says, “Where there are threats of serious or irreversible damage, lack of full scientific certainty shall not be used as a reason for postponing cost-effective measures to prevent environmental degradation.”

Note that the “Rio Declaration” calls for “cost-effective measures”, which suggests a similar weighing of risks and benefits as the recommendation to keep doses “as low as reasonably achievable, taking into account economic and societal factors” (see above). Admittedly, not every version of the ‘precautionary principle’ contains this qualification. Quite often, the emphasis is very much on avoiding risks. This is perhaps the main reason why the ICRP preferred prudence as a core value: it contains the notion of a careful consideration of both, the negative as well as the positive consequences of an action or practice. In a way, it is precaution combined with solidarity, if by the latter we understand (for want of a better term) “taking into account economic and societal consequences”.

As this brief discussion also indicates, the precautionary principle is not beyond criticism, and may need further explication (Hansson 2020). That does not mean, however, that its substance would be controversial. In our context it may mean this: those in charge of setting the rules of radiological protection cannot excuse themselves on the grounds of uncertainties in our scientific knowledge; they have to act upon plausible indications of risks, while not losing sight of the reasonability of their actions, taking into account economic and societal factors. This requires critical evaluation of the existing evidence, as well as exercising their responsibilities in terms of the “core values” mentioned above. The ICRP itself (ICRP_138 2018) has put it as follows: “Neither prudence nor the precautionary principle should be interpreted as demanding zero risk, choosing the least risky option, or requiring action just for the sake of action. The experience of over half a century of radiological risk management applying the optimisation principle can be considered as a reasoned and pragmatic application of prudence and/or the precautionary principle”. It is not impossible, of course, that at some point a revision of the system of radiological protection will become necessary, perhaps even a reassessment of the LNT model, but that must be left to rational analysis and discussion and cannot be pushed through by sowing doubts regarding the scientific anchoring of radiological protection as it is practised now.

## Conclusions

There is no doubt that, in order to be accepted by stakeholders and society at large, the system of radiological protection must be based on solid science. A common misconception, however, is that only conclusions that can be positively “proven” experimentally or epidemiologically are “scientific”. Notably, the assumption of direct proportionality with radiation dose for certain health effects (the linear no-threshold model) has been called unscientific because results describing effects after very low doses are inconclusive. Here we argue that it is not positive “proof” which renders a hypothesis “scientific”, but its fundamental “testability”. Currently, direct evidence in support of the LNT model is available down to a few tens of mSv. Testing it at even smaller doses seems possible in principle, but such studies are not available yet and must be left for the future. In situations like this, ethical considerations take on special importance - which does not render the whole system unscientific either. The precautionary principle, in particular, can certainly be critiqued and its application in radiological protection discussed as part of a rational endeavour to improve the practice of radiological protection, but it is an essential component of the core value of prudence which can be understood as “precaution tempered with solidarity”. Thus, attempts to discredit the LNT approach as being non-scientific lack any sound basis, and are in fact counterproductive with respect to the aims of radiological protection, because they preclude any constructive debate.

## Data Availability

No datasets were generated or analysed during the current study.
